# Irisin Ameliorate Acute Pancreatitis and Acinar Cell Viability through Modulation of the Unfolded Protein Response (UPR) and PPARγ-PGC1α-FNDC5 Pathways

**DOI:** 10.3390/biom14060643

**Published:** 2024-05-30

**Authors:** Avital Horwitz, Ruth Birk

**Affiliations:** Nutrition Department, Faculty of Health Sciences, Ariel University, Ariel 40700, Israel; avitalho@ariel.ac.il

**Keywords:** exocrine pancreas, pancreatitis, cerulin, irisin, ER stress, ATF6, XBP-1

## Abstract

Acute pancreatitis (AP) entails pancreatic inflammation, tissue damage and dysregulated enzyme secretion, including pancreatic lipase (PL). The role of irisin, an anti-inflammatory and anti-apoptotic cytokine, in AP and exocrine pancreatic stress is unclear. We have previously shown that irisin regulates PL through the PPARγ-PGC1α-FNDC5 pathway. In this study, we investigated irisin and irisin’s pathway on AP in in vitro (AR42J-B13) and ex vivo (rat primary acinar) models using molecular, biochemical and immunohistochemistry methodology. Pancreatitis induction (cerulein (cer)) resulted in a significant up-regulation of the PPARγ-PGC1α-FNDC5 axis, PL expression and secretion and endoplasmic reticulum (ER) stress unfolded protein response (UPR) signal-transduction markers (CHOP, XBP-1 and ATF6). Irisin addition in the cer-pancreatitis state resulted in a significant down-regulation of the PPARγ-PGC1α-FNDC5 axis, PPARγ nucleus-translocation and inflammatory state (TNFα and IL-6) in parallel to diminished PL expression and secretion (in vitro and ex vivo models). Irisin addition up-regulated the expression of pro-survival UPR markers (ATF6 and XBP-1) and reduced UPR pro-apoptotic markers (CHOP) under cer-pancreatitis and induced ER stress (tunicamycin), consequently increasing cells viability. Irisin’s pro-survival effect under cer-pancreatitis state was abolished under PPARγ inhibition. Our findings suggest irisin as a potential therapeutic option for AP via its ability to up-regulate pro-survival UPR signals and activate the PPARγ-PGC1α-FNDC5 pathway.

## 1. Introduction

Acute pancreatitis (AP) is a severe temporary inflammatory condition of the exocrine pancreas, characterized by the rapid acinar cells injury triggered by premature digestive enzymes activation, consequently resulting in autodigestion of the pancreatic parenchyma. AP represents a significant medical concern, with a prevalence of approximately 13–45/100,000 individuals annually worldwide [1]. In the United States, AP is one of the most common gastrointestinal conditions leading to hospital admission [2]. In severe AP, patients may present with systemic manifestations necessitating urgent medical intervention [3]. The pathophysiology of AP is complex and remains incompletely understood, yet recent studies have shown that the occurrence and development of AP are closely related to endoplasmic reticulum (ER) stress at the cellular and biological levels [4,5,6]. 

The ER serves a multitude of functions vital for the synthesis, folding and processing of secretory and transmembrane proteins [7]. Given the specialized role of pancreatic acinar cells in the high rate of digestive enzymes synthesis, an abundant ER presence is imperative to sustain the required and proper protein synthesis rate [8]. In some pathological conditions such as pancreatitis, the ER function is disrupted, resulting in the accumulation of misfolded and unfolded proteins in the ER lumen, leading to ER stress. Cellular ER stress state triggers the activation of highly conserved adaptive signal transduction known as the unfolded protein response (UPR), acting to restore ER homeostasis [9,10]. Upon detection of ER stress, three main transmembrane proteins—PERK (protein kinase RNA-like ER kinase), IRE1 (inositol-requiring enzyme 1) and ATF6 (activating transcription factor 6)—are activated to initiate UPR signaling pathways [11]. PERK phosphorylates eIF2α (eukaryotic initiation factor 2α) leads to the global translation attenuation and selective translation of ATF4 (activating transcription factor 4), which regulates genes involved in apoptosis, amino acid metabolism and antioxidant responses [12]. IRE1 activation splices XBP1 (X-box binding protein 1) mRNA, yielding an active transcription factor that up-regulates ER chaperones and ER-associated degradation (ERAD) components, promotes the restoration of ER homeostasis and cells survival [13]. ATF6 is translocated to the Golgi apparatus and undergoes a proteolytic cleavage to release a transcriptionally active fragment (cleaved ATF6) that translocates to the nucleus and acts as a transcription factor, promoting the expression of ER chaperones, folding enzymes and XBP-1, facilitating the restoration of cellular homeostasis [14,15,16]. Chronic or unresolved ER stress can contribute to cell dysfunction and augmented apoptosis, implicating ER stress and UPR dysregulation in various diseases, including pancreatitis [4,7]. For example, it was shown that ER stress and the UPR play a role in the autophagy of pancreatic zymogen granules, aggravating the inflammatory response and inducing apoptosis of acinar cells under pancreatitis induction [17,18]. Yet, the molecular mechanisms linking AP and ER stress remain not fully understood. 

AP is marked by the early activation and release of digestive enzymes within pancreatic acinar cells, resulting in self-digestion of the gland and enzymes leakage into the bloodstream [19]. One of the main digestive enzymes is pancreatic lipase (PL; triacylglycerol lipase EC 3.1.1.3), which is involved in a wide array of metabolic pathways, including lipid digestion, absorption, fatty acid uptake, lipoprotein transformation and inflammation [20]. PL is regulated by both the amount and type of dietary fat, but the mechanism is not fully understood [21,22,23]. 

PPARγ, a member of the PPARs family, functions as a ligand-activated transcription factor involved in regulating numerous metabolic processes, particularly related to lipid and glucose homeostasis [24]. Following interaction with the specific ligands, PPARγ (complexed with RXR) translocates to the nucleus, recruits co-activators such as peroxisome proliferator-activated receptor Gamma coactivator 1 (PGC-1) and binds to the peroxisome proliferator response element (PPRE) gene promoter, leading to the regulation of gene transcription [25]. PPARγ-identified ligands are a variety of natural or synthetic lipophilic acids, particularly fatty acids or their derivatives [26]. Given their diverse effects on glucose and lipid metabolism, as well as cell proliferation and apoptosis, PPARs and their modulators have been proposed as potential treatments for metabolic disorders like hyperglycemia and dyslipidemia. For instance, thiazolidinedione (TZD), an anti-diabetic medication, functions as a PPARγ agonist, enhancing insulin sensitivity, glucose tolerance and lipid balance [27]. We have recently showed that PPARγ directly regulates PL gene expression, possibly acting as the link between dietary fat and PL expression [28]. 

Irisin is a novel skeletal muscle-secreted myokine activated by proteolytic cleavage of the transmembrane protein fibronectin type III domain containing 5 (FNDC5) N-terminal domain (1–31 amino acids) [29]. Initial studies have shown irisin’s role in whole-body exercise-induced energy expenditure through white adipose tissue (WAT) browning and thermogenesis [30]. Subsequent studies have demonstrated irisin’s additional role in glucose and lipid metabolism, particularly in insulin resistance, diabetes, obesity and cardiovascular diseases [31,32,33]. Consequently, lower circulating levels of irisin were found to be associated with the onset of obesity and type 2 diabetes, nonalcoholic fatty liver disease and chronic kidney disease [31,34,35,36]. Recently, it was shown that irisin expression is regulated by fatty acids’ (FAs) stimuli, particularly saturated FAs (palmitic acid) in skeleton muscle and endocrine pancreas cells [37,38]. To date, irisin’s receptor has not been identified, and its signal transduction is mostly unknown. However, recently, the master regulator of metabolism, PGC1α, was found to regulate FNDC5-irisin synthesis in skeletal muscle in response to physical activity and various nutritional cues [39]. Furthermore, recently, it was indicated that irisin binds the αV class integrin in osteoclast and colon cells and mediates different cellular processes through the AMPK signal pathways [40,41]. Irisin’s protein sequence is well preserved across mammals, with 100% homology between human, rat and mouse [42]. We previously reported that FNDC5 is expressed in the exocrine pancreas and regulated by the PPARγ-PGC1α axis in response to dietary nutrients such as fatty acids, promoting PL expression and secretion. Moreover, exogenous irisin has an inhibitory effect on PL expression and secretion via negative feedback on the expression and activation of the PPARγ-PGC1α-FNDC5 pathway [43]. Irisin is expressed in both the endo and the exocrine pancreas. However, irisin’s role in the pancreas is not fully known, particularly in the exocrine pancreas. Irisin’s role in the pancreas was initially focused on the endocrine pancreas, glucose homeostasis and the influence on endocrine pancreas pathologies such as type 2 diabetes [44]. We previously reported a crosstalk between endocrine and exocrine pancreas pathologies [45], and irisin was shown to be involved in exocrine pancreas stress, AP and mitochondrial dysfunction in acinar cells [46]. However, the mechanisms underlying the role of irisin in the exocrine pancreas pathologies and ER stress are unknown. The aim of this research was to study the effect of irisin on ER stress levels under acute pancreatitis. 

## 2. Materials and Methods

### 2.1. Cell Culture

The AR42J-B13 cells (kindly provided by Dr I. Kojima, Gunma University, Maebashi, Japan) were maintained and differentiated as previously described [43]. 

### 2.2. Primary Pancreatic Acinar Cells

Male Sprague–Dawley rats (n = 8) were purchased from Envigo (Jerusalem, Israel) and treated according to the Ariel University Animal Ethics Committee (ethics approval IL-2206-121). Rat primary acinar cells were isolated and cultured as previously described by Gout et al. [47]. Twenty-four hours after isolation, primary acinar cells were counted as previously described [45], and an equal number of acini were transferred into a new culture dish for further secretion experiments as previously described [43].

### 2.3. Experimental Protocol

Pancreatitis state stimulation was induced using cerulin (cer; 100 nM; Sigma-Aldrich, Jerusalem, Israel), which was added to differentiated cells for 24 h. Exogenous irisin (60 ng/mL; Peprotech, Rehovot, Israel) was added to the differentiated cells for 4 h. ER stress was induced using tunicamuycin (TM; 5 μg/mL; Sigma-Aldrich, Jerusalem, Israel), added to differentiated cells for 6 h. The specific antagonist of PPARγ 3335 (32 μM; Sigma-Aldrich, Jerusalem, Israel) was added to differentiated cells for 24 h. For secretion experiments, an equal number of cells was grown in minimal medium without FBS for the indicated time points and treatments. 

### 2.4. Protein Extraction

Protein extraction was performed according to standard techniques, using RIPA lysis buffer as previously described [43]. Protein concentration was measured using a Bradford assay (Bio-Rad, Rison-Lezion, Israel) [48]. 

### 2.5. Western Blot Analysis

Equal concentrations of protein samples were prepared and further loaded on a 10–20% SDS polyacrylamide gel followed by transfer to nitrocellulose membrane as previously described [43]. Densitometry analysis of immunoblots was performed using ImageJ software version 1.4. All proteins were quantified relative to housekeeping protein actin or GAPDH. 

### 2.6. RNA Isolation and cDNA Synthesis

RNA was isolated using standard laboratory techniques as previously described [43]. Total RNA was reverse transcribed into cDNA using Tetro RT Enzyme, Random Hexamer Primer Mix and dNTP Mix (Tetro, Bioline, Bnei-Zion, Israel) according to the manufacturer’s protocol as previously described [43].

### 2.7. Quantitative RT-PCR (qPCR)

Transcript levels were determined by qPCR using SYBRs Green PCR Master Mix (Life Technologies, Rhenium, Modi’in-Maccabim-Re’ut, Israel) as previously described [43]. 

### 2.8. Immunocytochemistry (ICC) Staining

AR42J-B13 cells were grown and differentiated on cover slips and were subject to the different treatments as indicated in the experimental protocol section. Cells were fixed, permeabilized and blocked as previously described [43], following by overnight incubation with primary rabbit antibodies against PPARγ (Abcam, Tel-Aviv, Israel). Primary antibodies were detected using 1 h incubation with goat anti-rabbit-IgG coupled to Alexa Fluor 488 (Abcam, Israel). Nuclear labeling was performed with DAPI (Sigma-Aldrich, Israel). Immunofluorescence staining was visualized using an Olympus microscope, X81 (Eisenberg Bros. Ltd., Modi’in-Maccabim-Re’ut, Israel)and the quantification of PPARγ nuclear localization was conducted as previously described [43].

### 2.9. Statistical Analysis

Statistical analysis was performed using GraphPad Prism software (version 8). Data are expressed as mean ± SD from at least 3 independent experiments. Comparisons between control and treatments (cer, irisin, TM, combined treatments) were made using one-way ANOVA followed by Tukey’s post hoc test. 

## 3. Results

### 3.1. Pancreatitis Induction (Cer-Treatment) Activate the PPARγ-PGC1α-FNDC5 Pathway and Induce PL Expression and Secretion

In order to confirm cer-pancreatitis mimicking effect, the influence of cer treatment on the inflammatory status and viability of acinar cells was explored in differentiated AR42J-B13 treated with 100 nM cer for 6, 18 and 24 h. Cer-pancreatitis induction resulted in a significant elevation of the inflammation markers TNFα and IL-6, accompanied by the reduction in cell viability in a time-dependent manner (Supplementary Figures S1 and S2), indicating that prolonged (24 h) cer treatment mimic AP state in vitro. 

**Figure 1:**
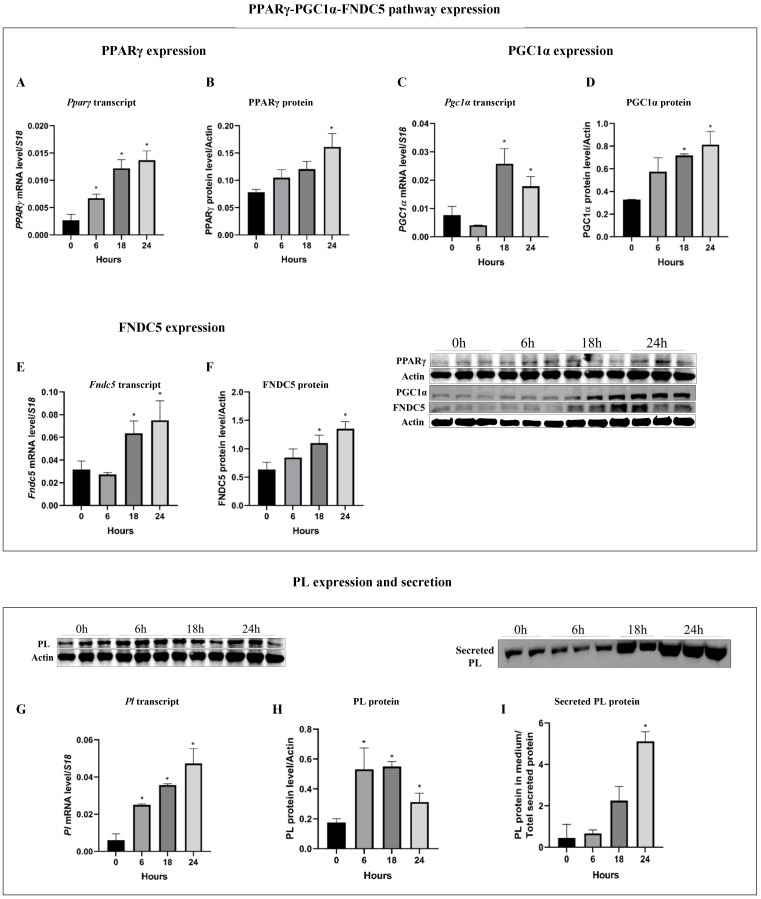
The PPARγ-PGC1α-FNDC5 pathway was activated and up-regulated PL expression and secretion in response to cerulin treatment. AR42J-B13 cells were differentiated for 48 h and subject to cerulin treatment (100 nM) for 6, 18 and 24 h. Total RNA and protein were extracted during differentiation and subjected to RT-qPCR and western blot analysis, respectively. Expression levels were normalized to housekeeping gene *S18* and actin, for mRNA and protein, respectively. (**A**) *Pparγ* transcript levels, (**B**) PPARγ protein levels, (**C**) *Pgc1α* transcript levels, (**D**) PGC1α protein levels, (**E**) *Fndc5* transcript levels, (**F**) FNDC5 protein levels, (**G**) *Pl* transcript levels, (**H**) PL protein levels, (**I**) secreted PL protein levels. Results are expressed as mean ± SE of 3 independent experiments (n = 3). * Asterisks represent statistical difference from control (*p* < 0.05). Original images can be found in Supplementary Materials.

We previously showed that exocrine pancreas PL expression and secretion is regulated by the PPARγ-PGC1α-FNDC5 pathway. Next, we aimed to explore whether pancreatitis induction affects the PPARγ-PGC1α-FNDC5 axis and PL expression and secretion. Under cer treatment, PPARγ protein and transcript levels were significantly and time-dependently higher, reaching a maximal elevation of 2-fold (protein) and 5.6-fold (transcript) after 24 h of cer treatment compared to untreated cells at time 0 (Figure 1A,B). Similarly, PGC1α levels were significantly elevated in response to 18 h and 24 h of cer treatment, by 3.4-fold (transcript) and 2.2-fold (protein), and by 2.3-fold (transcript) and 2.5-fold (protein), respectively (Figure 1C,D). Cer treatment significantly up-regulated FNDC5 levels starting at 18 h of cer treatment by 1.6-fold (transcript) and 1.8-fold (protein) and by 1.9-fold (transcript) and 2.1-fold (protein) after 24 h (Figure 1E,F). PL transcript and protein levels were significantly up-regulated by 5.5-fold (transcript) and by 1.6-fold (protein) after 6 h of cer treatment, and they reached a maximal significant elevation of 9-fold (transcript) and 3-fold (protein) after 18 h compared to the control untreated cells (Figure 1G,H). Accordingly, cer treatment significantly enhanced the secretion of PL by 2.5-fold compared to untreated cells after 18 h and reached a maximal up-regulation of secreted levels of 4.8-fold after 24 h compared to untreated cells (Figure 1I). Together, these results indicate that pancreatitis induction up-regulates the expression and secretion of PL suggestively via the activation of the PPARγ-PGC1α-FNDC5 pathway.

### 3.2. Irisin Dulls the Increase in PPARγ-PGC1α-FNDC5 Axis and PL Production under Cer-Pancreatitis Conditions

**Figure 2:**
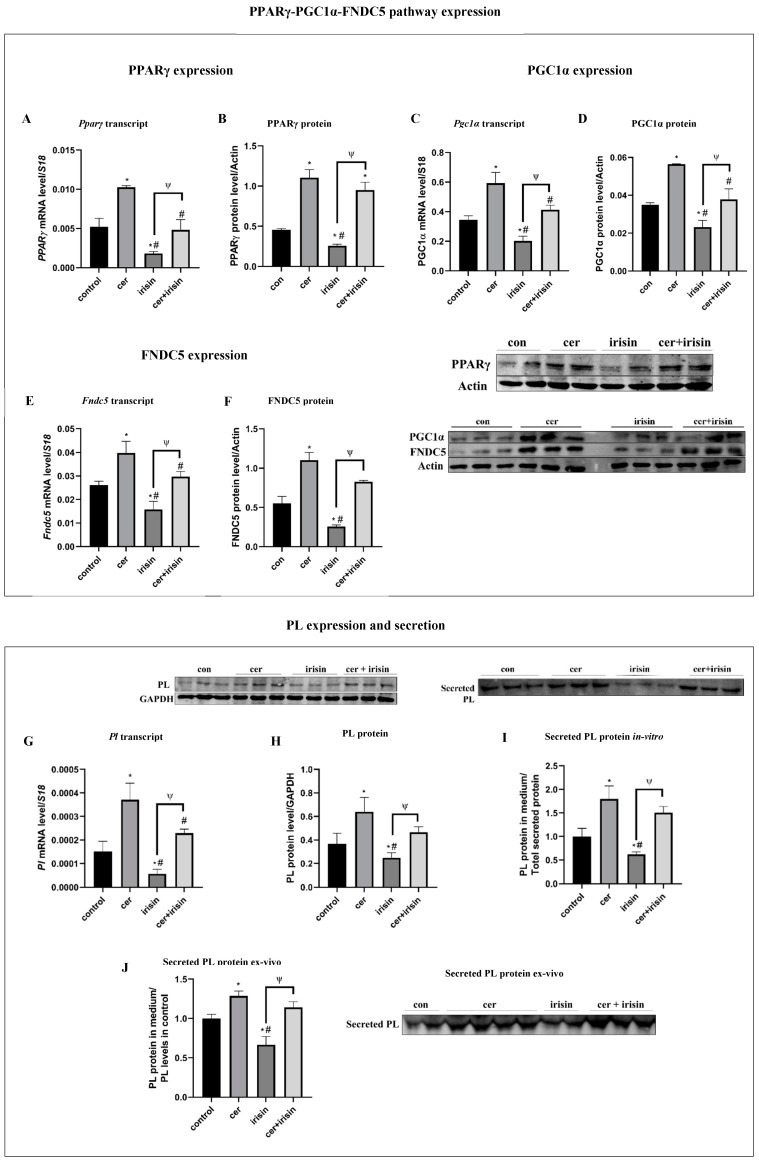

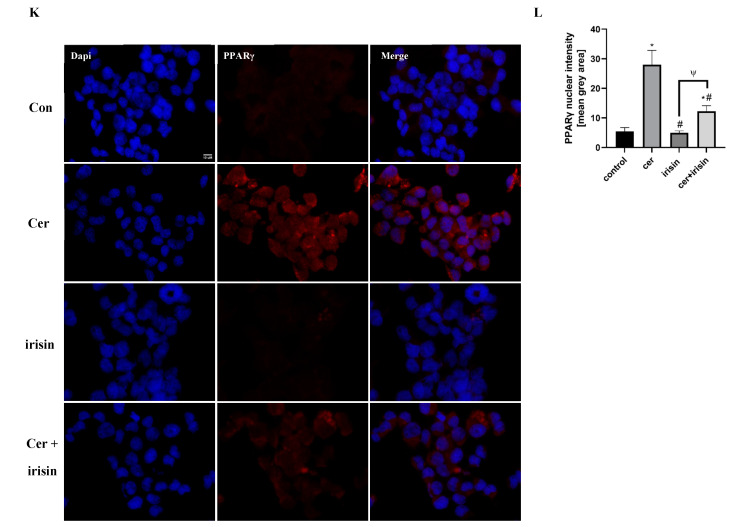
Irisin abolished the up-regulation of PPARγ-PGC1α-FNDC5 pathway and PL expression and secretion under cer-pancreatitis treatment. AR42J-B13 cells were subject to cer (100 nM, 24 h) exogenous irisin (60 ng/mL, 4 h) or combed treatments for 24 h. Total RNA and protein were extracted during differentiation and subjected to RT-qPCR and western blot analysis, respectively. Medium was collected and subjected to western blot analysis. Expression levels were normalized to housekeeping gene *S18* for mRNA and actin or GAPDH for protein, respectively. Primary exocrine pancreas acinar cells were subject to cer (100 nM, 24 h) exogenous irisin (60 ng/mL, 4 h) or combed treatments for 24 h, and the medium was collected and subjected to western blot analysis. (**A**) *Pparγ* transcript levels, (**B**) PPARγ protein levels, (**C**) *Pgc1α* transcript levels, (**D**) PCG1α protein levels, (**E**) *Fndc5* transcript levels, (**F**) FNDC5 protein levels, (**G**) *Pl* transcript levels, (**H**) PL protein levels, (**I**) secreted PL protein. (**J**) Ex-vivo secreted PL protein in primary rat acinar cells. (**K**) Immunohistochemistry staining was performed on differentiated acinar cells following treatment with cer (24 h) or irisin (4 h) and visualized by immunofluorescence labeling and fluorescence microscopy by anti-PPARγ primary antibody (red) followed by goat anti-rabbit IgG Alexa Fluor 488 and by DAPI nucleus staining (blue). Immunohistochemistry photos represent fields of cells (n = 1000–1500); clusters were randomly selected from triplicates of two independent experiments. Photos were taken using Olympus fluorescent microscope at ×60 magnification. (**L**) Quantification of PPARγ nuclear localization was performed using ImageJ software (n = 200–250 for each treatment). Results are expressed as mean ± SE of 3 independent experiments (n = 3). * Asterisks represent statistical difference from control (*p* < 0.05), # represents statistical difference (*p* < 0.05) from cer, ψ represents statistical difference (*p* < 0.05) from irisin. Original images can be found in Supplementary Materials.

Pancreatitis is characterized by a significant elevation in the expression and secretion of digestive enzymes. We previously showed that exogenous irisin have a suppressive effect on PL expression and secretion [43]. Next, we aimed to study the combined effect of cer and irisin treatments on the PPARγ-PGC1α-FNDC5 pathway. Cer-pancreatitis significantly increased PPARγ levels compared to control levels (transcript and protein levels by 1.3 and 1.4-fold, respectively). In contrast, exogenous irisin treatment significantly decreased PPARγ levels by 2.6-fold (transcript) and by 1.4-fold (protein) compared to untreated control cells. The combination of cer and irisin treatment restored Pparγ transcript levels to a similar level measured in the control, while PPARγ protein levels remain like PPARγ levels of measured in cer-treated cells (Figure 2A,B). PGC1α levels were significantly down-regulated in irisin-treated cells by 1.7-fold (transcript) and by 1.5-fold (protein) compared to the control and were significantly increased in response to cer-pancreatitis by 1.7-fold (transcript) and by 1.6-fold (protein). Cer + irisin treatment restored PGC1α levels (both transcript and protein) to similar levels as untreated cells. However, when compared to cer treatment alone, the combination cer + irisin treatment significantly reduced PGC1α transcript levels by 1.5-fold (transcript) and by 2-fold (protein) compared to cer-treated cells (Figure 2C,D). FNDC5 levels were significantly higher under cer treatment by 1.5-fold (transcript) and by 2-fold (protein) and were significantly reduced by 1.6-fold (transcript) and by 2.1-fold (protein) in response to exogenous irisin compared to the control. FNDC5 transcript and protein levels under the cer + irisin treatment were significantly higher only when compared to irisin-treated cells by 1.9-fold and by 3-fold, respectively (Figure 2E,F). Moreover, cer and irisin had an opposite effect on PL levels, where cer treatment significantly up-regulated PL expression by 17-fold (transcript) and by 1.4-fold (protein), and irisin treatment significantly reduced PL expression by 1.6-fold (protein) and by 3.6-fold (transcript). The combined cer + irisin treatment reduced PL protein levels similar to PL protein expression levels in control (Figure 2G,H). The combined cer + irisin treatment had a suppressor effect on PL secretion levels, resulting in a significant down-regulation of secreted PL levels by 1.4-fold compared to cer-treated cells and by 2.4-fold compared to irisin treatment alone (Figure 2I). To further establish irisin’s initiatory effect on PL secretion, secreted PL levels were measured in rat primary acinar cells, an acceptable model for digestive enzymes’ secretion. Similarly, to secreted PL levels in the in vitro model, cer treatment significantly enhanced secreted PL protein levels by 1.3-fold and by 1.9-fold compared to the control and exogenous irisin treatment, respectively. In contrast, exogenous irisin treatment significantly reduced secreted PL levels by 1.5-fold compared to untreated cells (Figure 2J). PPARγ acted as a nuclear receptor as well as a transcription factor. Upon activation, PPARγ translocated to the nucleus and stimulated gene expression, and PPARγ’s presence in the nucleaus indicated the activation of PPARγ and PPARγ-altered signal transduction. We previously reported that irisin had an inhibitory effect on the translocation of PPARγ to the nucleus and that exogenous irisin had an inhibitory effect on the PPARγ-PGC1α pathway. In acinar cells treated with cer, PPARγ (red) was translocated to the nucleus, indicating a significant activation of the PPARγ-PGC1α pathway. In contrast, exogenous irisin treatment significantly decreases PPARγ translocation to the nucleus, indicating that exogenous irisin has an inhibitory effect on the PPARγ-PGC1α pathway (Figure 2K,L).Together, our findings demonstrate that cer-pancreatitis treatment significantly up-regulates the PPARγ-PGC1α-FNDC5 pathway, in parallel PL expression and secretion (Figure 1 and Figure 2), while exogenous irisin had an inhibitory effect on PPARγ-PGC1α-FNDC5 and PL.

### 3.3. Irisin Treatment Suppresses Inflammation Markers following Pancreatitis

**Figure 3:**
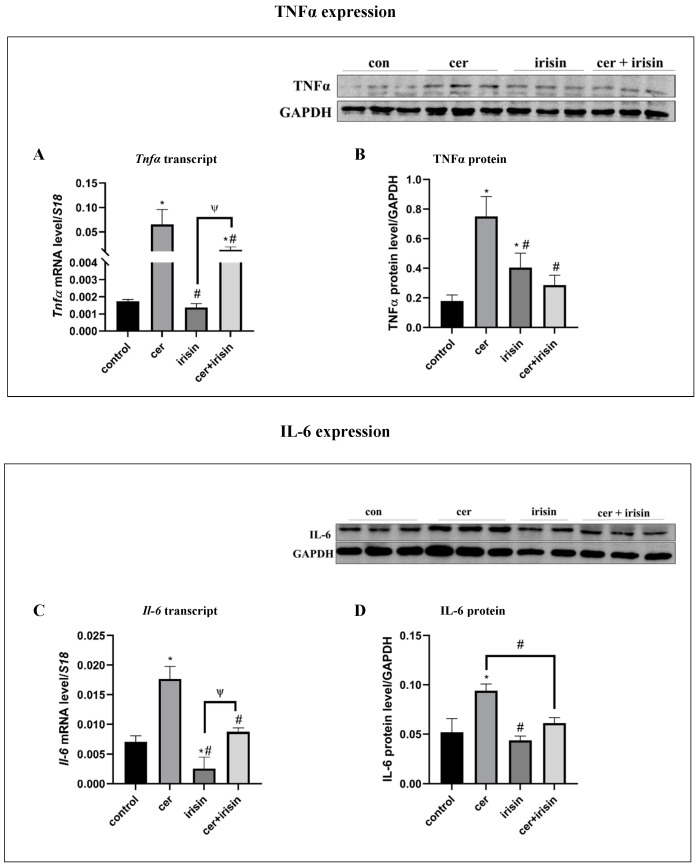
Irisin amended the inflammation status in cer-pancreatitis cells. AR42J-B13 cells were subjected to cer (100 nM, 24 h) exogenous irisin (60 ng/mL, 4 h) or combed treatments. Total RNA and protein were extracted during differentiation and subjected to RT-qPCR and western blot analysis, respectively. Expression levels were normalized to housekeeping gene *S18* (mRNA) and actin or GAPDH (protein) respectively. (**A**)* Tnfα* transcript levels, (**B**) TNFα protein levels, (**C**) *Il-6* transcript levels, (**D**) IL-6 protein levels. Results are expressed as mean ± SE of 3 independent experiments (n = 3). * Asterisks represent statistical difference from control (*p* < 0.05), # represents statistical difference (*p* < 0.05) from cer, ψ represents statistical difference (*p* < 0.05) from irisin. Original images can be found in Supplementary Materials.

Cer-pancreatitis resulted in a significant up-regulation of inflammation markers such as TNFα and IL-6 (Supplementary Figure S2). Next, we aimed to investigate the effect of exogenous irisin on the inflammatory status in the cells. Cer-pancreatitis significantly elevated TNFα expression levels by 36-fold (transcript) and by 4-fold (protein). The combined cer + irisin treatment significantly down-regulated TNFα expression by 4.9-fold (transcript) and by 2.5-fold (protein) compared to cer-treated cells (Figure 3A,B). Similarly, IL-6 levels were significantly elevated under cer-pancreatitis by 2.5-fold (transcript) and by 1.7-fold (protein) compared to the control, while the addition of exogenous irisin significantly reduced IL-6 expression levels by 2-fold (transcript) and by 1.4-fold (protein) compared to cer-pancreatitis alone (Figure 3C,D). 

### 3.4. Irisin Treatment Suppresses Anti-Survival URP Agents Parallel to Activation of Pro-Survival URP Agents under Cer-Pancreatitis Treatment

**Figure 4:**
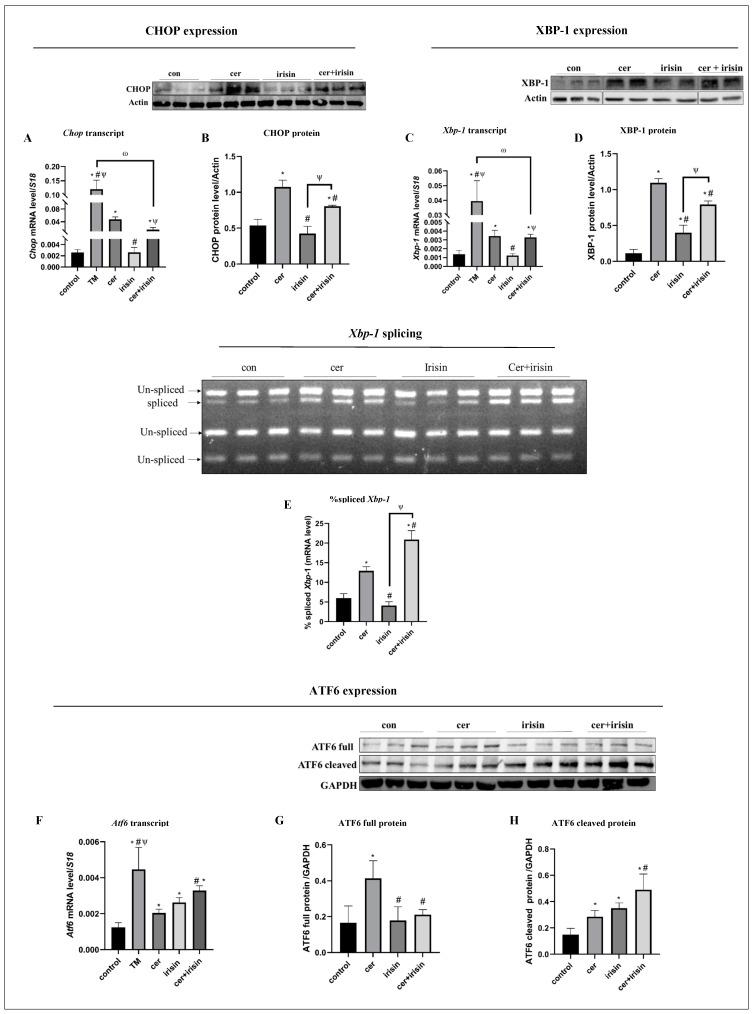
Irisin up-regulated pro-survival UPR agents and down-regulated anti-survival UPR agents under cer-pancreatitis conditions. AR42J-B13 cells were subject to cer (100 nM, 24 h) exogenous irisin (60 ng/mL, 4 h) or combined treatments. Total RNA and protein were extracted and subjected to RT-qPCR and western blot analysis, respectively. Expression levels were normalized to housekeeping gene *S18* gene (mRNA) and actin or GAPDH (protein). The percentage of *Xbp-1* splicing was calculated as the intensity of sXbp-1 divided by total intensities of *Xbp-1* (*sXbp-1* and *uXbp-1*). (**A**) *Chop* transcript levels, (**B**) CHOP protein levels, (**C**) *Xbp-1* transcript levels, (**D**) XBP-1 protein levels, (**E**) quantification of %*sXbp-1*, (**F**) *Atf6* transcript levels, (**G**) ATF6 full protein levels, (**H**) ATF6 cleaved protein levels. Results are expressed as mean ± SE of 3 independent experiments (n = 3). * Asterisks represent statistical difference from control (*p* < 0.05), # represents statistical difference (*p* < 0.05) from cer, ψ represents statistical difference (*p* < 0.05) from irisin, ω represents statistical difference (*p* < 0.05) from TM. Original images can be found in Supplementary Materials.

As we have previously shown, cer-pancreatitis induced acinar cells’ ER stress [49]. Thus, we explored the ER stress status in response to irisin. As expected, using ER stress inducer TM resulted in ER stress and UPR activation expressed by significant up-regulation of Xbp-1, Chop and Atf6 transcript levels by 37-, 3.2- and by 25-fold, respectively, compared to the control (Figure 4A,C,F). Similarly, cer-pancreatitis induction significantly up-regulated the CHOP levels by 7.3-fold (transcript) and by 2-fold (protein), XBP-1 levels by 3.2-fold (transcript) and by 9.7-fold (protein) and Atf6 by 1.6-fold (transcript) compared to the control (Figure 4A–D). The spliced active form sXbp-1 levels were significantly higher in cer-treated cells by 2.4-fold compared to the control (Figure 4E). Irisin supplementation alone did not significantly affect CHOP expression levels (Figure 4A,B). In contrary, the combined cer-irisin treatment significantly reduced CHOP levels by 1.5-fold (transcript) and by 1.3-fold (protein) compared to cer-treated cells (Figure 4A,B) and restored the total Xbp-1 transcript levels to similar levels of the control cells (Figure 4C). Yet, cer + irisin treatment significantly increased XBP-1 protein levels by 7-fold compared to control and by 2-fold compared to irisin treatment (Figure 4D). Additionally, the combined treatment further elevated the active sXbp-1 levels by 4.4-fold compared to control and by 1.8-fold compared to cer-treated cells (Figure 4D). Irisin treatment elevated Atf6 transcript levels by 2-fold compared to control and by 1.3-fold compared to cer-treated cells. The cer-irisin treatment further elevated Atf6 transcript levels by 2.7-fold compared to the control and by 1.6-fold compared to cer-treated cells (Figure 4F). ATF6 full protein level was significantly up-regulated in response to cer treatment by 2.5-fold and by 2.3-fold compared to control and irisin-treated cells, respectively. Also, the active cleaved ATF6 protein was significantly up-regulated in cer-treated cells by 2-fold compared to untreated cells (Figure 4G,H). Interestingly, while irisin supplementation did not affected the ATF6 full protein expression, it had a significant positive effect on ATF6 protein processing, up-regulated the cleaved ATF6 protein levels by 2.3 and 1.2-fold compared to control and cer-treated cells, respectively. Moreover, the combined cer + irisin treatment further elevated the ATF6 cleaved protein expression by 3.2- and 1.7-fold compared to untreated and cer treatment cells, respectively (Figure 4G,H). Together, these results indicate that exogenous irisin specifically activates pro-survival UPR agents such as XBP-1 and ATF6. 

### 3.5. Irisin Tilts the Apoptosis Status towards Survival and Improved Cells Viability under Cer-Pancreatitis Condition

**Figure 5:**
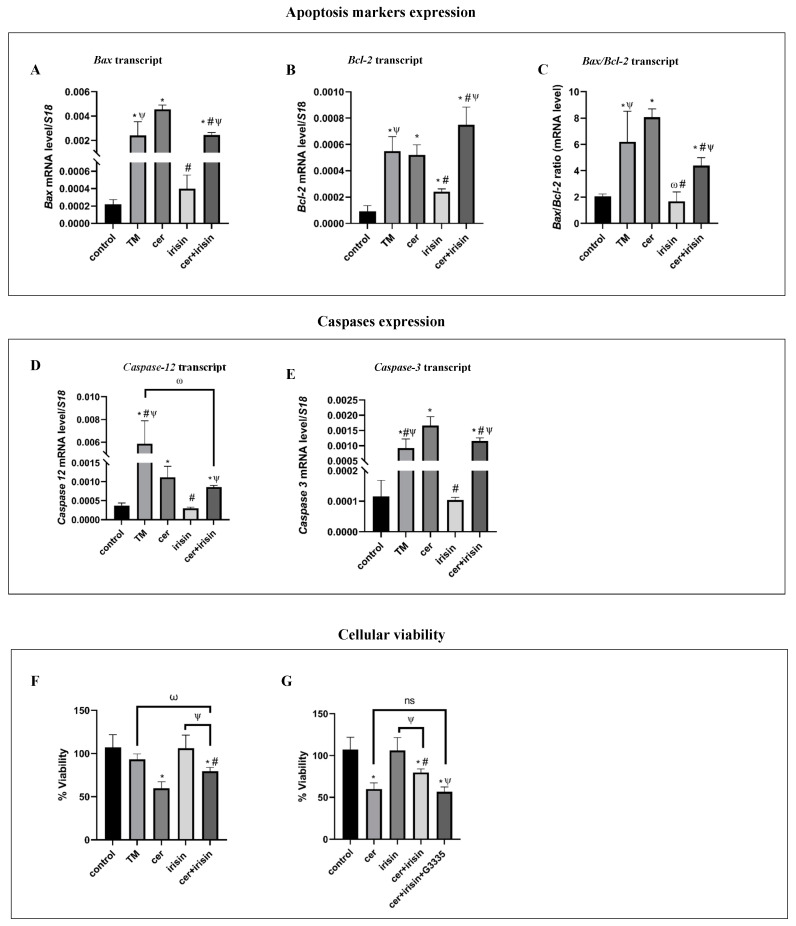
Combination of irisin and cer treatments reduced apoptosis markers level and improved cells viability. AR42J-B13 cells were subject to cer (100 nM, 24 h) exogenous irisin (60 ng/mL, 4 h) or combined treatments. Total RNA and protein were extracted during differentiation and subjected to RT-qPCR analysis. Expression levels were normalized to housekeeping gene *S18*. For % viability analysis, AR42J-B13 cells were differentiated for 48 h, seeded on 12-well plate (500,000 cells/mL) and treated with TM (5 μg/mL; 6 h) cerulin (100 nM; 24 h), irisin (60 ng/mL; 4 h), the combined cerulin (100 nM; 24 h) + irisin (60 ng/mL; 4 h) and G3335 (32 μM; 24 h). Viable and dead cells were distinguished by trypan blue exclusion test. (**A**) *Bax* transcript levels, (**B**) *Bcl-2* transcript levels, (**C**) *Bax/Bcl-2* ration, (**D**) *Caspase-12* transcript levels, (**E**) *Caspase*-3 transcript levels, (**F**) % viability under cer + irisin treatments, (**G**) % viability under cer + irisin treatment with the addition of PPARγ agonist G3335. Results are expressed as mean ± SE of 3 independent experiments (n = 3). * Asterisks represent statistical difference from control (*p* < 0.05), # represents statistical difference (*p* < 0.05) from cer, ψ represents statistical difference (*p* < 0.05) from irisin, ω represents statistical difference (*p* < 0.05) from TM. ns represent non-statistical difference.

Cer-pancreatitis induction resulted in a significant reduction in cell viability after 24 h of cer treatment (Supplementary Figure S1E,F). In contrast, exogenous irisin up-regulated the expression of pro-survival UPR proteins (Figure 4), implying that irisin might influence acinar cells survival. Next, we evaluated the apoptosis status under cer and irisin treatment. Bax transcript levels were significantly up-regulated following cer-treatment by 20- and 11.4-fold compared to the control and irisin treatment, respectively. The addition of exogenous irisin to cer-treated cells led to a significant reduction in Bax transcript levels by 1.5-fold compared to cer treatment alone. However, cer-irisin treatment did not reduce the levels to reach control levels (Figure 5A). Bcl-2 transcript levels were significantly raised in both TM and cer-treated cells by 5.2- and 3.7-fold, respectively, compared to the control. Irisin treatment alone significantly increased the transcript levels of Bcl-2 by 2.6-fold compared to the control. Cer-irisin treatment resulted in a significant up-regulation of Bcl-2 transcript levels by 8.9-, 1.7-, and 2.4-fold compared to control, cer-treated and irisin-treated cells (Figure 5B). The Bax/Bcl-2 ratio was significantly higher in cer-treated cells by 3.9-fold and 4.9-fold compared to control and irisin treatment, respectively. The Bax/Bcl-2 ratio measured in cer + irisin-treated cells were significantly higher by 2.1-fold compared to the control, yet the Bax/Bcl-2 ratio was significantly reduced by 1.8-fold compared to cer treatment alone (Figure 5C). Caspase 12 is a member of the caspase family, localized to the ER membrane and is activated specifically in response to prolonged ER stress, playing a crucial role in initiating apoptosis under conditions of severe ER dysfunction [50]. Caspase 12 transcript levels were significantly elevated by 3.5- and 20-fold in cer-treated and TM-treated cells, respectively, compared to the control, suggesting the involvement of ER stress-mediated apoptosis induction in response to prolonged cer treatment. In contrast, irisin treatment or the combined cer-irisin treatment did not affect Caspase 12 transcript levels, compared to untreated cells and cer-treated cells, respectively (Figure 5D). Caspase-3 is a crucial enzyme involved in the execution phase of apoptosis, responsible for the cleavage of various cellular substrates leading to cell dismantling and programmed cell death [51]. The transcript expression levels of Caspase-3 were significantly up-regulated in cer-treated cells (by 14.5-fold compared to the control), indicating activation of apoptosis pathways. In contrast, the addition of irisin had a significant protective effect, reducing the Caspase-3 transcript expression measured in cer + irisin-treated cells by 2.2-fold compared to cer-treated cells (Figure 5E). The apoptosis status measured following cer treatment resulted in a significant reduction in cells % viability by 1.8-fold compared to both untreated cells and irisin-treated cells, which showed comparable viability. The addition of irisin to cer-treated cells resulted in a significantly higher % viability by 1.4-fold compared to cer treatment alone (Figure 5F). To verify the role of the PPARγ-PGC1α-FNDC5 axis in the improvement in % viability under the combined cer and exogenous irisin treatment, we treated the cells with the highly specific PPARγ antagonist G3335 (32 μM). The addition of G3335 abolished the positive effect of irisin in the combined cer + irisin + G3335 treatment, resulting in a significant reduction by 1.9-fold compared to levels of both untreated and irisin treated cells. Moreover, the % viability measured in the cer + irisin + G3335-treated cells did not significantly differ from cer treatment alone (Figure 5G), suggesting the improvement in % viability under cer + irisin treatment is related to PPARγ activity.

### 3.6. The PPARγ-PGC1α-FNDC5 Pathway Is Activated in ER Stress Condtions and Inhibited in Response to Exogenous Irisin

**Figure 6:**
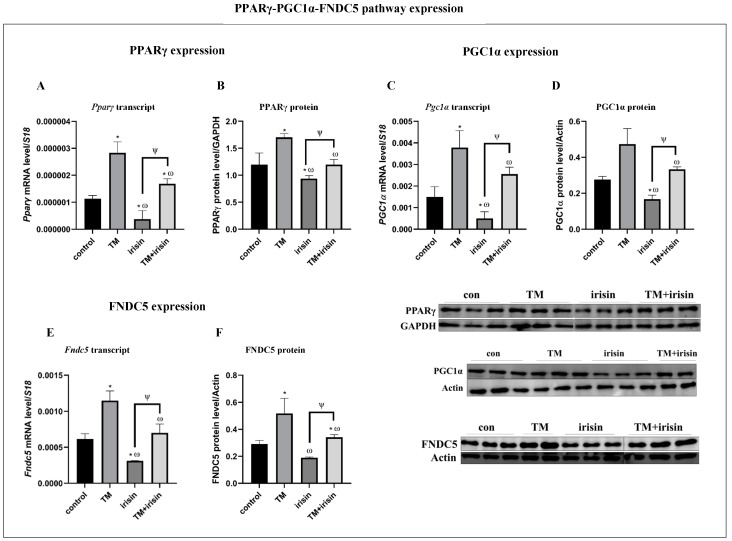
TM treatment activated the PPARγ-PGC1α-FNDC5 axis in contra to irisin’s inhibitory effect. AR42J-B13 cells were subject to TM (5 μg/mL, 6 h) exogenous irisin (60 ng/mL, 4 h) or the combined treatments. Total RNA and protein were extracted during differentiation and subjected to RT-qPCR and western blot analysis, respectively. Expression levels were normalized to housekeeping gene *S18* (mRNA) and actin or GAPDH (protein), respectively. (**A**) *Pparγ* transcript levels, (**B**) PPARγ protein levels, (**C**) *Pgc1α* transcript levels, (**D**) PCG1α protein levels, (**E**) *Fndc5* transcript, levels, (**F**) FNDC5 protein levels. Results are expressed as mean ± SE of 3 independent experiments (n = 3). * Asterisks represent statistical difference from control (*p* < 0.05), ω represents statistical difference (*p* < 0.05) from TM, ψ represents statistical difference (*p* < 0.05) from irisin. Original images can be found in Supplementary Materials.

Cer-pancreatitis activated the PPARγ-PGC1α-FNDC5 pathway (Figure 1 and Figure 2) and triggered the activation of the UPR. In addition, exogenous irisin supplementation activated pro-survival UPR agents under cer-pancreatitis conditions and improvement in % viability. Moreover, inhibition of PPARγ activity abolished the positive effect of irisin on cer-treated cells (Figure 4 and Figure 5). To understand if ER stress is linked to irisin signal transduction pathway, we exposed acinar cells to an ER stress state combined with exogenous irisin. Similar to pancreatitis induction, TM treatment significantly elevated the expression of the PPARγ-PGC1α-FNDC5 pathway (Figure 6); the expressions of PPARγ and PGC1α were significantly elevated under ER stress conditions compared to the control cells (PPARγ transcript and protein expression by 2.5-fold and by 1.4-fold, respectively; PGC1α transcript and protein expression by 2-fold and by 1.7-fold, respectively) (Figure 6A–D). Likewise, FNDC5 expression was up-regulated in TM-treated cells by 1.8-fold (transcript and protein) compared to the control. The addition of irisin to the TM treatment significantly down-regulated Pparγ transcript levels by 1.5-fold and PPARγ protein levels by 1.4-fold compared to TM treatment alone (Figure 6A,B). Correspondingly, the combination of TM and irisin treatment resulted in a significant reduction in PGC1α expression by 1.4-fold (transcript and protein) compared to TM treatment alone (Figure 6C,D) and significantly reduced FNDC5 expression by 1.5-fold (transcript) and by 16-fold (protein) (Figure 6E,F). Together, these results indicate the involvement of the PPARγ-PGC1α-FNDC5 in cellular stress conditions such as pancreatitis and the ER stress state.

### 3.7. Irisin Spacificaly Activae Pro-Survival UPR Agents under ER Stress Conditions

**Figure 7:**
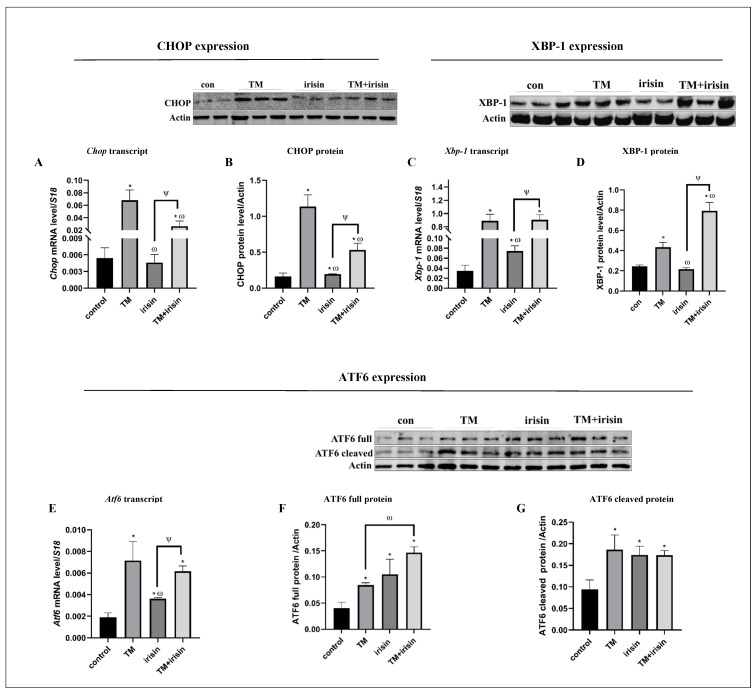
Irisin up-regulates pro-survival-UPR activation under ER stress induction in acinar cells. AR42J-B13 cells were subject to TM (5 μg/mL, 6 h) exogenous irisin (60 ng/mL, 4 h) or the combined treatments. Total RNA and protein were extracted during differentiation and subjected to RT-qPCR and western blot analysis, respectively. Expression levels were normalized to housekeeping gene *S18* and actin, for mRNA and protein, respectively. (**A**) *Chop* transcript levels, (**B**) CHOP protein levels, (**C**) *Xbp-1* transcript levels, (**D**) XBP-1 protein levels, (**E**) *Atf6* transcript levels, (**F**) ATF6 full protein levels, (**G**) ATF6 cleaved protein levels. Results are expressed as mean ± SE of 3 independent experiments (n = 3). * Asterisks represent statistical difference from control (*p* < 0.05), ω represents statistical difference (*p* < 0.05) from TM, ψ represents statistical difference (*p* < 0.05) from irisin. Original images can be found in Supplementary Materials.

Irisin supplementation mediated ER stress levels and UPR activation under the pancreatitis mimicking state. Next, we aimed to explore whether irisin addition has a similar effect on the modulation of the different UPR branches under a specific ER stressor treatment. Irisin’s addition to ER stress-induced cells had a similar effect on ER stress markers expression as seen in cer + irisin-treated cells (Figure 4). CHOP levels were significantly reduced in TM + irisin-treated cells by 2.5-fold (transcript) and by 1.3-fold (protein) compared to TM treatment alone (Figure 7A,B). Additionally, and like XBP-1 expression pattern in cer + irisin-treated cells (Figure 4C,D), the combination TM + irisi treatment resulted in a significant elevation in XBP-1 levels by 26-fold and by 3.2-fold (transcript and protein, respectively) compared to the control. Xbp-1 transcript levels were similar between TM and TM + irisin-treated cells, while XBP-1 protein levels were significantly higher under the combined TM + irisin treatment by 1.7-fold compared to TM treatment (Figure 7C,D). ATF6 expression and processing under TM + itisin treatment also had a similar pattern as under the combined cer + irisin treatment (Figure 4F–H). Atf6 transcript levels were significantly up-regulated by 4.2- and 1.9-fold in response to TM and irisin treatment, respectively, compared to the control cells. The combined TM + irisin treatment further increased Atf6 transcript levels by 3.5- and 1.8-fold compared to the levels in the control and irisin-treated cells, respectively (Figure 7E). ATF6 full protein levels significantly increased by all treatments (TM, irisin, TM + irisin) by 2.2-, 2.7- and 3.4-fold, respectively, compared to the control. Also, the combination of TM + irisin significantly elevated the ATF6 full protein expression by 1.5- and 1.3-fold, respectively, compared to the levels measured in response to the TM and irisin treatment separately (Figure 7F). The cleaved active form of ATF6 was significantly up-regulated in response to the treatment of TM, irisin and TM + irisin by 1.7-fold compared to control (Figure 7G). These results further indicate that irisin has a positive effect on pro-survival UPR agents under ER stress induction.

## 4. Discussion

We demonstrated that irisin mitigated pancreatitis stress via up-regulating pro-survival UPR signals. Exogenous irisin had an inhibitory effect on the PPARγ-PGC1α-FNDC5 pathway, mediating reduction in PL synthesis and secretion. AP is a sudden inflammatory condition marked by pancreatic tissue damage and stress [1]. Irisin was shown to have an anti-inflammatory, anti-apoptotic and anti-oxidative properties in several tissues, including the endocrine pancreas [52,53,54]. We and others have shown that exocrine pancreas stress, e.g., ER stress in acinar cells is a prerequisite in the development of pancreatic pathologies [10,45]. Moreover, we previously reported the role of the FNDC5-PGC1α-PPARγ pathway in the regulation of PL expression and secretion [43], which is disrupted in the AP state. Our current research studied the influence of irisin on the cellular and molecular mechanisms underlying AP and exocrine pancreatic stress using in vitro and ex vivo models. In vitro and ex vivo models, particularly rodent models induced by cer, mimicked key aspects of human acute pancreatitis, aiding in the investigation of pathophysiological processes. Cer, a peptide analog of the hormone cholecystokinin (CCK), serves as a potent pharmacological agent for inducing experimental pancreatitis in research models [55]. Molecularly, AP involves intracellular events such as activation of inflammatory pathways, including TNFα and IL-6, which play a key role in the inflammatory response [56,57]. TNFα, a pro-inflammatory cytokine, contributes to pancreatic tissue injury through the induction of oxidative stress, leukocyte infiltration and endothelial dysfunction [58]. It was found that the levels of TNFα measured in the pancreas were directly related to the severity of pancreatic damage [59]. Furthermore, blood TNFα concentrations increased after AP induction [60]. IL-6, synthesized by various cell types including pancreatic acinar cells, plays a multifaceted role in AP, promoting inflammation, tissue damage and the systemic acute-phase response [61]. In line with these pro-stress effects, we demonstrated that prolonged cer-pancreatitis led to a significant reduction in acinar cells viability, accompanied by a gradual elevation in the expression of TNFα and IL-6 in response to pancreatitis-mimicking induction in vitro (Supplementary Figures S1 and S2). Both TNFα and IL-6 are implicated in the regulation of apoptotic and necrotic pathways, influencing the balance between cell survival and death in the pancreas during acute pancreatitis [62,63,64]. The dysregulation of digestive enzymes activation, including PL, within the pancreatic acinar cells is pivotal in disease progression [65,66]. Therefore, the inhibition of expression and secretion of pancreatic enzymes during AP can reduce the severity of AP and allow the rehabilitation of the exocrine pancreas. As can be seen in Figure 1, cer treatment activated the FNDC5-PGC1α-PPARγ pathway, resulting in an intensified PL expression and secretion in a time-dependent manner. We reported that irisin had an inhibitory effect on the expression and secretion of PL in acinar cells via the negative effect on the FNDC5-PGC1α-PPARγ pathway [43], suggesting that exogenous irisin might have a beneficial effect on the acinar cells under AP conditions. The addition of exogenous irisin to cer-treated cells resulted in a significant reduction in the expression of the FNDC5-PGC1α-PPARγ axis (Figure 2A–F), possibly through a negative feedback regulation, resulting in reduced acinar cells functionality and pancreatic lipase production. Additionally, exogenous irisin reduced the translocation of activated PPARγ to the nucleus (Figure 2K,L). PPARγ acts in the nucleus as a transcription factor to up-regulate the transcription of various target genes, including PL [28]. Thus, the negative effect of irisin on PPARγ expression and activation resulted in the down-regulation of PL expression and secretion (Figure 2G–I). These results were repeated in an ex vivo model of primary acinar cells, an acceptable model of pancreatic enzymes secretion, where the exogenous irisin supplementation reduced the secreted PL levels (Figure 2J). Additionally, and in parallel the reduction in the FNDC5-PGC1α-PPARγ axis expression and activation, exogenous irisin seems to have a beneficial effect on the inflammatory state in the AP-induced acinar cells. Exogenous irisin treatment significantly reduced the high expression of TNFα and IL-6 in cer-treated acinar cells (Figure 3). Several studies have reported an anti-inflammatory effect of exogenous irisin in various cell types. For example, the supplementation of exogenous irisin suppressed the expression of pro-inflammatory genes and reduced cellular apoptosis in rat INS-1E β cells [67]. Together, these results indicate that the inhibitory effect of exogenous irisin on the FNDC5-PGC1α-PPARγ pathway causes a decrease in PL expression and secretion under AP induction, an underlying mechanism for the development and aggregation of AP. The reduction in acinar cells activity under the supplementation of irisin was parallel to the improvement in the inflammatory state of the cer-treated cells. We previously reported the involvement of ER stress and UPR activation in exocrine pancreas acinar cells subjected to AP induction [45]. Moreover, several studies have reported that irisin can attenuate ER stress and ER stress-induced apoptosis in different cell types such as hepatocytes and cardiomyocytes [68,69,70]. Additionally, exogenous irisin affected ER stress and UPR activation in the endocrine pancreas related pathologies [70,71]. We previously showed a crosstalk between endocrine and exocrine pancreas pathologies, with specificity in the activation of pro-survival and pro-apoptotic UPR agents [45]. Furthermore, it was reported that exogenous irisin treatment improved the survival and pancreatic injury in L-arginine-pancreatitis-induced mice, parallel to the reduction of several ER stress markers such as GRP78 and p-IRE1α [46]. AP is characterized by high ER stress levels, possibly due to an augmented expression and secretion on digestive enzymes [5,72]. Considering the inhibitory effect of irisin on PL expression and secretion (Figure 2G–J), we aimed to explore the effect of irisin supplementation on ER stress and UPR activation in cer-treated acinar cells. Cer treatment significantly elevated ER stress levels and triggered the UPR activation reflected by the up-regulation of the UPR agents CHOP (Figure 4A,B), XBP-1 (Figure 4C–E) and ATF6 (Figure 4F–H). The ability to respond to perturbations in ER function is a fundamentally important property of all cells, but ER stress can also lead to apoptosis. The activation of each branch of the UPR leads to slightly different pro-survival or pro-apoptotic responses of the UPR, and cell fate is determined by the balance between pro-survival and pro-apoptotic signals. Yet, the molecular mechanisms that facilitate this switch are not fully understood [73,74]. Out of the UPR agents, CHOP is an important element of the switch from pro-survival to pro-death signaling and is well known to promote apoptotic cell death [75,76,77]. CHOP causes changes in gene expression that favor apoptosis, including increasing the expression of Bim and Bax and decreasing the expression of Bcl-2 [78,79]. Accordingly, the expression of the apoptotic marker *Bax* and the *Bax/Bcl-2* ratio was significantly elevated in cer-treated cells (Figure 5A,C), as well as *caspase 3* expression (Figure 5E), indicating a shift towards apoptosis initiation under AP-state induction, reflected by a significant reduction in acinar cells viability in response to cer treatment (Supplementary Figure S1F and Figure 5F,G). However, irisin supplementation significantly diminished the cer-induced elevation in CHOP expression levels (Figure 4A,B), a similar pattern as that observed in TM + irisin treated acinar cells (Figure 7A,B). The significant down-regulation in CHOP expression in response to irisin addition was accompanied by a significantly moderate decrease in cell mortality in cer + irisin-treated cells compared to cer-treated cells (Figure 5F,G), suggesting irisin influences the balance between pro- and anti-apoptotic signals. In addition, exogenous irisin up-regulated the expression of ATF6, especially the cleaved active form of ATF6 (Figure 4F–H and Figure 7E–G). Upon ER stress induction, ATF6 translocates to the Golgi apparatus where it is cleaved into its active form which then moves to the nucleus and induces pro-survival genes, including XBP-1 [80,81]. ATF6-mediated signals seem to be purely pro-survival and aim to counteract ER stress [73]. The addition of exogenous irisin to cer or TM treatments resulted in a significant up-regulation of the ATF6 transcript and protein (Figure 4F–H and Figure 7E–G), suggesting irisin acts to activate the pro-survival UPR branch under stress conditions (e.g., AP induction). In parallel, a significant up-regulation and activation of XBP-1 was observed in response to the combination of stress (cer and TM) and irisin treatment (Figure 4C–E and Figure 7C,D). ATF6 was shown to promote the expression and activation of XBP-1 [81]. The spliced form of XBP-1 induces the expression of a large number of genes involved in almost all aspects of the UPR upgrading cellular survival through the induction of ER chaperones and protein degradation [82]. Several studies have reported that ATF6 and XBP-1 act together to promote cell survival. For example, it was shown that ATF6 heterodimerizes with other UPR factors, such as XBP-1, acting together to increase the expression of UPR target genes, such as chaperon proteins, XBP-1 and GRP78, in order to restore the proteomic balance and maintain cellular homeostasis, and the knockdown of ATF6 markedly reduced ATF6-pathway target gene expression, including XBP-1 [83,84]. Together, it may imply that the improvement in cell viability observed in the combination cer + irisin treatment is related to an intensified activation of pro-survival pathways during UPR activation. We showed that PPARγ acts as a regulator FNDC5 expression in the exocrine pancreas [43], and our results suggest that exogenous irisin plays role in the shift towards survival in the balance between survival and apoptosis under AP conditions (Figure 4, Figure 5 and Figure 7). The positive effect of irisin on cell viability under AP induction was thwarted by the specific PPARγ antagonist G3335 (Figure 5F), further indicating that the activation of PPARγ, possibly via the PPARγ-PGC1α-FNDC5 pathway, is related to the improvement in cell viability under exogenous irisin supplementation. A few studies reported irisin beneficial effect on apoptotic status in several cells, including endocrine pancreas cells [53,85,86]. The positive effect of irisin on the cellular viability observed in our study (Figure 5F–G) might be downstream of the PPARγ-PGC1α-FNDC5 pathway, due to the modulation of pro- and anti-apoptotic markers activated under stress conditions in favor of pro-survival agents. Similar to our results, several studies have shown that exogenous irisin enhanced the expression of the pro-survival agent Bcl-2, parallel to reducing the anti-survival agents expression such as Bax and Caspase-3 [87,88,89]. Additionally, irisin might influence cellular stress and ER stress through the down-regulation of UPR markers, resulting in the modulation of pro- and anti-apoptotic balance. Thus, irisin’s effect on acinar cells viability suggestively is through both pathways’ modulation, resulting in crosstalk of the pro- and anti-apoptotic balance. Furthermore, preliminary research has reported a possible crosstalk between the PPARγ and UPR pathways [90]; however, more research is needed on this subject.

## 5. Conclusions

In conclusion, AP mimicking in acinar cells resulted in a significant reduction in cell viability accompanied by inflammatory markers’ elevation and an ER stress state in a time-dependent manner. Additionally, cer subjection activated the PPARγ-PGC1α-FNDC5 pathway in acinar cells, as well as augmented PL expression and secretion. Exogenous irisin had an inhibitory effect on the PPARγ-PGC1α-FNDC5 axis, and reduced PL expression and secretion in both in vitro and ex vivo models. Additionally, exogenous irisin had a protective effect against inflammation and ER stress-induced apoptosis, possibly via the influence on the up-regulation and activation of pro-survival signals such as cleaved ATF6 and XBP-1, leading to decreased apoptosis in stress acinar cells treated with exogenous irisin. The beneficial effect of irisin supplementation was abolished with the blockage of PPARγ activity, further indicating the involvement of the PPARγ-PGC1α-FNDC5 axis in the modulation of UPR activation characterized to AP. In parallel, PL synthesis and secretion were inhibited, contributing to the diminished cellular stress. Taken together, our results suggest that irisin has a pro-survival effect on AP-inflicted acinar cells, expressed in the activation of the pro-survival UPR signal transduction, parallel to the inhibition of anti-survival pathways. Irisin supplementation altered the PPARγ-PGC1α-FNDC5 axis, leading to a reduction in PL synthesis and secretion. Thus, exogenous irisin holds promise as a potential therapeutic agent for AP.

## Data Availability

The original contributions presented in the study are included in the article/Supplementary Materials, further inquiries can be directed to the corresponding authors.

## Supplementary Materials

The following supporting information can be downloaded at: https://www.mdpi.com/article/10.3390/biom14060643/s1, Figure S1: AR42J-B13 morphology and viability in response to cerulin treatment; Figure S2: Inflammation markers in response to cerulin treatment. File S2: Western Blotting Figures.
