# Supplementary material for: Irisin Ameliorate Acute Pancreatitis and Acinar Cell Viability through Modulation of the Unfolded Protein Response (UPR) and PPARγ-PGC1α-FNDC5 Pathways
Source: Biomolecules. 2024 May 30;14(6):643. doi: 10.3390/biom14060643 (PMC11201894; doi:10.3390/biom14060643)
Supplement: Supplementary file 1 [file biomolecules-14-00643-s001.zip › Supplementary Figure S1.pdf]

Supplementary Figure S1:

Time scale of cerulin effect on acinar cells morphology and viability

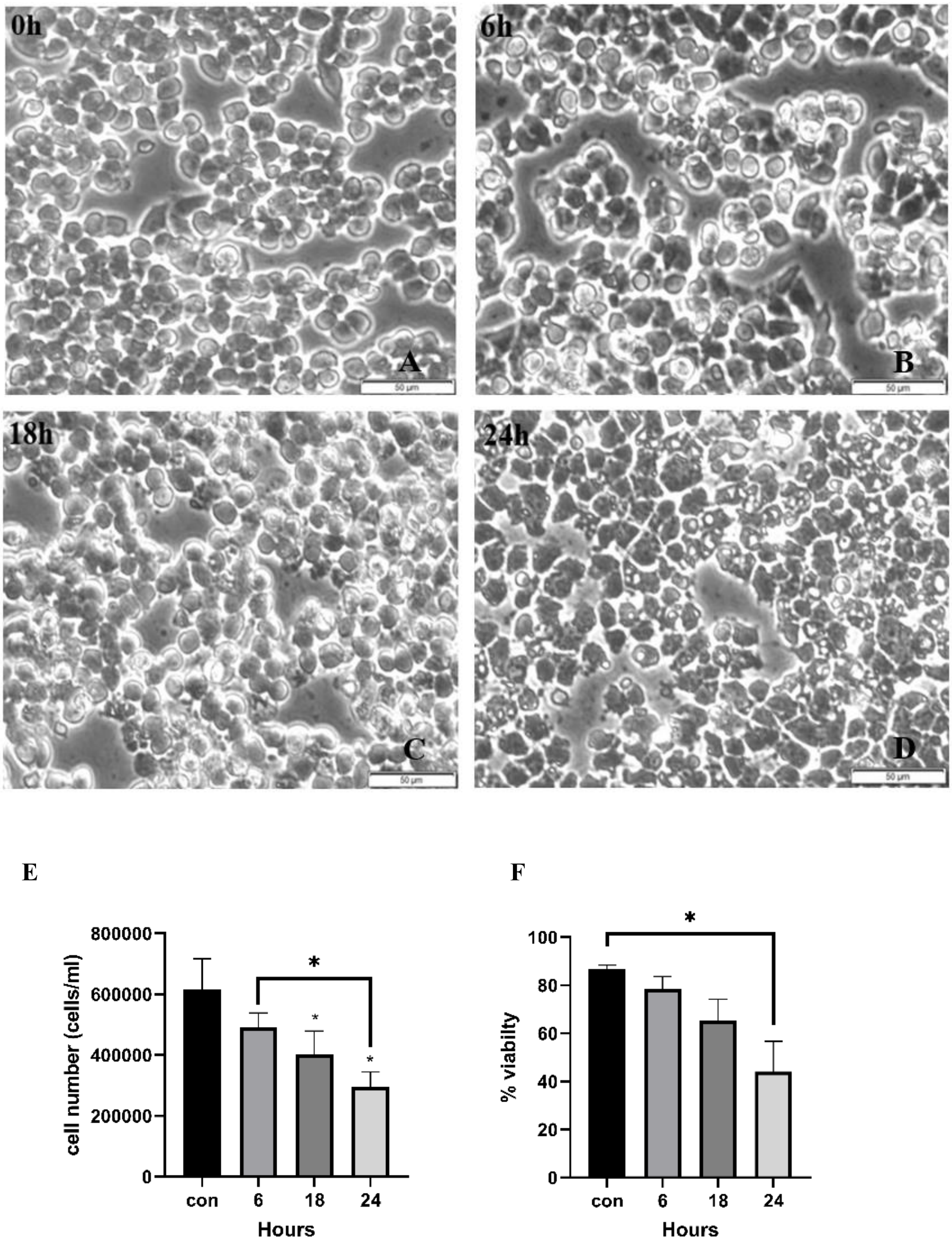

Supplementary Figure 1: AR42J-B13 morphology and viability in response to cerulin treatment. AR42J-B13 cells were differentiated for 48hr, seeded on 12-well plate (500,000 cells/ml) and treated with cerulin (100nM) for 6, 18 and 24hr. Cells were observed under light microscopy (Olympus X81) at X20 magnitude and photographed at the indicated time points **(A)** Time 0

(baseline) **(B)** Time 6hr **(C)** Time 18hr **(D)** Time 24hr. Viable and dead cells were distinguished by trypan blue exclusion test. **(E)** Cell number (cells/ml) **(F)** % viability. Results are expressed as the mean ± SE of 3 independent experiments (n=3). * Asterisks represent statistical difference ($P<0.05$).
