# Supplementary material for: Irisin Ameliorate Acute Pancreatitis and Acinar Cell Viability through Modulation of the Unfolded Protein Response (UPR) and PPARγ-PGC1α-FNDC5 Pathways
Source: Biomolecules. 2024 May 30;14(6):643. doi: 10.3390/biom14060643 (PMC11201894; doi:10.3390/biom14060643)
Supplement: Supplementary file 1 [file biomolecules-14-00643-s001.zip › Supplementary Figure S2.pdf]

Supplementary Figure S2:

Inflammation markers in cerulin-treated cells

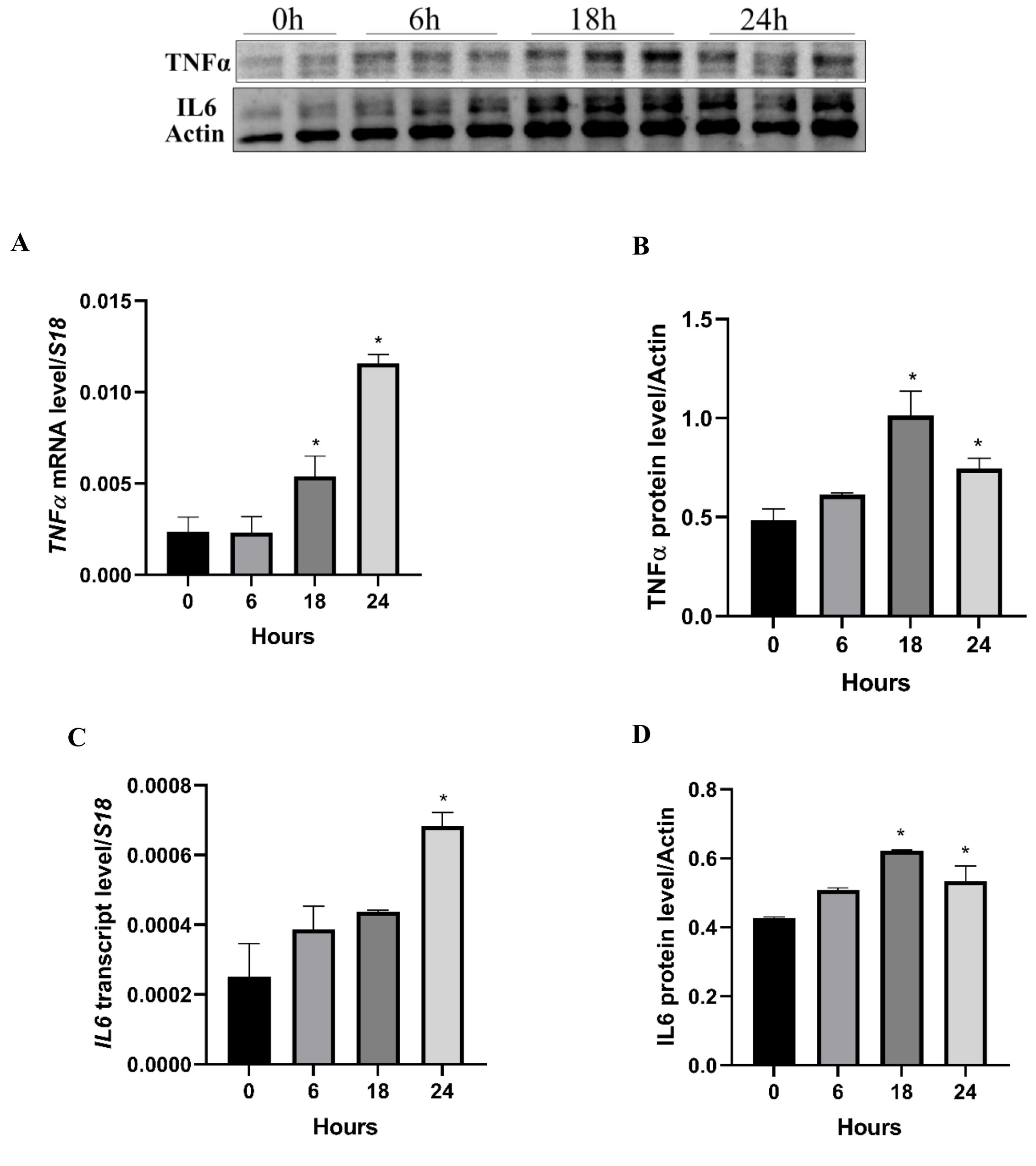

Supplenemtary Figure 2: Inflammation markers in response to cerulin treatment. AR42J-B13 cells were differentiated for 48hr and subject to cerulin treatment (100nM) for 6, 18 and 24hr. Total RNA and protein were extracted during differentiation and subjected to RT-qPCR and western blot analysis, respectively. Expression levels were normalized to housekeeping gene S18 and actin, for mRNA and protein, respectively. **(A)** *Tnfα* transcript levels **(B)** TNFα protein levels **(C)** *Il-6* transcript levels **(D)** IL-6 protein levels. Results are expressed as mean

±SE of 3 independent experiments (n=3). *Asterisks represent statistical difference from time 0 ($P<0.05$).
